# Measuring acuity of the approximate number system reliably and validly: the evaluation of an adaptive test procedure

**DOI:** 10.3389/fpsyg.2013.00510

**Published:** 2013-08-06

**Authors:** Marcus Lindskog, Anders Winman, Peter Juslin, Leo Poom

**Affiliations:** Department of Psychology, Uppsala UniversityUppsala, Sweden

**Keywords:** approximate number system, adaptive measure, validity, reliability, ZEST

## Abstract

Two studies investigated the reliability and predictive validity of commonly used measures and models of Approximate Number System acuity (ANS). Study 1 investigated reliability by both an empirical approach and a simulation of maximum obtainable reliability under ideal conditions. Results showed that common measures of the Weber fraction (*w*) are reliable only when using a substantial number of trials, even under ideal conditions. Study 2 compared different purported measures of ANS acuity as for convergent and predictive validity in a within-subjects design and evaluated an adaptive test using the ZEST algorithm. Results showed that the adaptive measure can reduce the number of trials needed to reach acceptable reliability. Only *direct* tests with non-symbolic numerosity discriminations of stimuli presented simultaneously were related to arithmetic fluency. This correlation remained when controlling for general cognitive ability and perceptual speed. Further, the purported indirect measure of ANS acuity in terms of *the Numeric Distance Effect (NDE)* was not reliable and showed no sign of predictive validity. The non-symbolic NDE for reaction time was significantly related to direct *w* estimates in a direction contrary to the expected. Easier stimuli were found to be more reliable, but only harder (7:8 ratio) stimuli contributed to predictive validity.

## Introduction

Picture yourself hunting, slowly approaching a herd of gazelles on the African savanna. As you get closer the herd picks up your scent and scatters into two almost equally large groups that run off in different directions. To maximize your chance of a successful hunt, you quickly need to decide which of the two groups has more animals.

Together with infants and non-human animals, human adults share the ability to represent numerical magnitudes, for example the number of gazelle, without the use of symbols (Feigenson et al., 2004). The ability is supported by the Approximate Number System (ANS), which represents numbers and magnitudes in an analog and approximate fashion with representations becoming increasingly imprecise as numerosity increases (Dehaene, 2009; but see, Brannon et al., 2001).

The acuity of the ANS, how accurately it represents numerical magnitude, is conceptualized as the smallest change in numerosity that can be reliably detected by an individual. This acuity is often quantified by a Weber fraction (*w*). Recent studies have shown considerable individual variability in ANS acuity among humans (e.g., Pica et al., 2004; Halberda and Feigenson, 2008; Halberda et al., 2008; Tokita and Ishiguchi, 2010) and that ANS acuity improves (i.e., *w* decreases) developmentally from childhood to adolescence (Halberda and Feigenson, 2008).

Brain-imaging studies have indicated that the ANS has a neurological basis in the intraparietal sulcus (IPS) on the lateral surface of the parietal lobe (Castelli et al., 2006; Piazza et al., 2006). Studies on macaque monkeys have even found specialized neurons (numerons) within the IPS that are sensitive to numerosity (Nieder et al., 2002). The ANS is thought to be a fundamentally abstract representation independent of perceptual variables of lower order. This characteristic is essential because numerical quantity itself is a highly abstract concept [but see Gebuis and Reynvoet (2012) for an account that rejects the idea of an ANS altogether, and proposes that instead numerosity judgments stem from multiple weighted visual cues]. Further support for this independence from lower level perceptual variables in terms of supramodality was found in a recent study (Nieder, 2012a). Using single-cell recordings in the primate brain it was shown that there are neurons that encode numerosity irrespective of stimulus modality (visual/auditory) (see Nieder, 2012b for a review of the physiology of “number neurons”).

The IPS is activated when people attend to or compare the number of objects in a set, when they observe numbers in different modalities and when they perform simple arithmetic tasks (Piazza et al., 2004; Piazza and Izard, 2009). This suggests a relationship between ANS acuity and achievement on formal mathematical tasks. It has been proposed that the precision of the ANS and mathematical ability are associated due to a causal link from the former to the latter. The idea is that the neural correlates of the ANS lay the foundation for representations of symbolic higher-level arithmetical concepts. While such a relationship has been documented with children, even when controlling for a large number of cognitive abilities, (Halberda et al., 2008; Inglis et al., 2011) results from studies on adults are mixed (Gebuis and van der Smagt, 2011; Inglis et al., 2011; Castronovo and Göbel, 2012; Price et al., 2012).

One reason for the mixed results might be that different tasks have been used to measure both ANS acuity and mathematics achievement. Recently, the reliability and validity of some of the tasks most commonly used to measure ANS acuity have been challenged (e.g., Gebuis and van der Smagt, 2011; Gilmore et al., 2011; Price et al., 2012). In this article, we first review the inconclusive findings in regard to ANS acuity and mathematics achievement, suggesting that the current measures of ANS acuity may have limited reliability^1^. We thereafter validate this claim by reporting simulations that determine the upper limit of the reliability that can be achieved as a function of the length of the test. In view of these results we developed and tested an adaptive test of ANS acuity that allows valid and reliable measures of ANS acuity with a shorter test-length. This test also correlated significantly with measures of mathematical ability, also after controlling for more general cognitive abilities.

### ANS acuity and math achievement

Theories of magnitude representation (Feigenson et al., 2004; Dehaene, 2009) and brain imaging data (Nieder et al., 2002; Piazza et al., 2004, 2006; Dehaene, 2009; Nieder and Dehaene, 2009; Piazza and Izard, 2009) suggest that ANS acuity should be related to achievement in formal mathematical tasks. Such a relationship has been found all the way back to kindergarten mathematics performance in children (Halberda et al., 2008) when using a non-symbolic discrimination task with simultaneous presentation. Libertus et al. (2011) showed that the link is found prior to formal mathematics education. Desoete et al. (2012) showed that accuracy of non-symbolic judgments in kindergarten is related to arithmetical achievement in first grade. Mazzocco et al. (2011a) and Piazza et al. (2010) showed that impairment of the ANS is predictive of developmental dyscalculia.

With adults, the results are less clear-cut. Libertus et al. (2012) reported a correlation between ANS acuity and mathematics SAT scores with undergraduate students and Lyons and Beilock (2011) reported a correlation with mental arithmetic using university students. DeWind and Brannon (2012) obtained a correlation between self-reported math SAT/GRE scores and *w*. Lourenco et al. (2012) found a correlation between ANS acuity and Woodcock-Johnson calculation subtask that measures advanced arithmetic ability, as well as with a test of geometric knowledge, but failed to obtain correlations with a number of more elementary math subtasks. Surprisingly, this study also found significant correlations with judgments of cumulative area, rather than number.

Price and coworkers (Price et al., 2012) compared three different non-symbolic comparison tasks with both the numeric distance effect (NDE) and *w* as dependent variables, but found no correlations with arithmetic fluency (Woodcock et al., 2001) as the criterion variable. Castronovo and Göbel (2012) found that ANS precision did not correlate with math performance. Another recent study found a significant correlation for children but failed to do so for adults (Inglis et al., 2011). Inglis and colleagues (2011) suggested that the lack of relationship for adults indicates that the strength between the two constructs changes with age. They suggested that ANS plays a bootstrapping role in learning whole numbers but that other factors dominate how more sophisticated numerical concepts are learned (Inglis et al., 2011, p. 1228). The stimuli used by Inglis and colleagues, however, led both adults and children to rely on non-numerical visual cues to a large extent, resulting in 40% of the participants being excluded from the analysis. This may have affected the conclusions. It is possible, for example, that perceptual cue reliance is systematically related to performance.

Halberda et al. (2012) showed that ANS precision correlated with self-reported school mathematics ability with a sample of more than 10,000 participants, and across age groups from 11 to 85 years. This finding was replicated in a second study in the same paper with the presumably more valid and less vaguely defined self-reported scores on the mathematics subtest of the SAT. Another study showed that while performance on a discrimination task correlated with concurrent arithmetic fluency (addition), performance on a detection (same/different) task did not (Gebuis and van der Smagt, 2011).

The inconsistent results across studies with adults indicate that the task used to measure ANS acuity may influence the relationship with mathematical achievement. In the present study we further investigate the relationship between ANS acuity and arithmetical fluency by using both the task introduced by Halberda et al. (2008) and variants of this task. Use of a within-subjects design makes interpretation of results easier by eliminating variance due to different participant populations, different experimental procedures etc. We know of only one study (Price et al., 2012) that relies on a within-subjects procedure to study reliability and validity of different ANS-metrics and different tasks. We therefore used a within-subject design to be able to compare different measures of ANS acuity directly.

### Measuring ANS acuity

Measures of ANS acuity can be divided into *direct* and *indirect* measures, as discussed below. Common for these measures is that they are *static* in the sense that they present the same stimuli to all participants regardless of each participant's individual level of ANS acuity.

#### Direct measures

In the tasks that use direct measures of ANS acuity, the participants are either asked to *compare* two non-symbolic magnitudes (i.e., *which set is more numerous*) or to *detect* a change between two numerosities (*are these sets the same or different in numerosity?*). The latter detection task may either involve a baseline numerosity, which is constant from trial to trial, and a comparison numerosity that varies on each trial, or two stimuli that both vary from trial to trial. Most often the non-symbolic magnitudes are dot arrays presented briefly (around 200 ms) but other stimuli, for example arrays of squares, have also been used (e.g., Halberda et al., 2008; Maloney et al., 2010). Non-symbolic stimuli are thus sets of objects (e.g., five squares or three elephants) in contrast to symbolic stimuli where numerosity is represented by a numeric symbol (e.g., the Arabic digit 5 or the Roman numeral III). Direct measures of ANS acuity can also be classified into tasks with parallel and sequential stimulus presentation. In parallel tasks, which are predominant, both sets of non-symbolic magnitudes are presented at the same time, while sequential tasks display each set one at a time, often separated by a blank screen or a mask, where the inter-stimulus interval is often brief. The sequential presentation in detection tasks using baseline and comparison stimuli often presents the baseline numerosity several times before the comparison stimulus is presented (much like a habituation procedure). Simultaneously presented stimuli are most often spatially intermixed but sometimes they are separated spatially. When stimuli are presented spatially intermixed the areas occupied by the two sets are allowed to overlap (but no single element is allowed to overlap, or occupy the same space as, any other element) while there is no such overlap allowed for spatially separated stimuli.

#### Indirect measures

Indirect measures of ANS acuity attempt to measure effects that are, supposedly, consequences of ANS acuity rather than ANS acuity *per se*. For example, the *numeric distance effect* (NDE; Moyer and Landauer, 1967), where it takes longer for people to tell which of two digits is the larger when the digits are close on the number line (e.g., 5 and 6) than when the digits are further apart (e.g., 5 and 9), is considered to be a result of the way magnitudes are represented in the ANS (e.g., Dehaene, 1992). The rationale for using NDE as a measure of ANS acuity originates in the fact that both direct measures of ANS acuity and indirect measures produce a ratio effect where accuracy decreases and reaction time increases as the ratio between stimuli becomes larger. The NDE has been interpreted as a performance product of the noisiness of the representation of number, resulting in overlapping representations (Dehaene and Cohen, 1995, however, see Van Opstal et al., 2008 for an account of the distance effect that does not require overlapping representations). Consequentially, researchers have considered the NDE to be “a key metric of the ANS” (Price et al., 2012, p. 50) and used the size of the NDE as a measure of ANS acuity with larger NDE indicating larger w (e.g., Peters et al., 2008; Gilmore et al., 2011; Price et al., 2012). The NDE is sometimes also used as a measure with non-symbolic stimuli (e.g., Holloway and Ansari, 2009; Mundy and Gilmore, 2009).

The distinction made above between tests as *direct* and *indirect* is related to the implicit underlying validity of these tests. Direct tests have content validity in actually measuring behavioral performance directly related to the underlying construct, whereas indirect tests can be viewed as relying on construct validity, related to theoretical assumptions and models. It is thus fully possible that an individual with a large NDE is better at discrimination between any pair of numerosities (both in terms of accuracy and reaction times) than another individual with a small NDE.

#### Dependent measures of ANS acuity

The tasks described above generally produce one, or more, of three types of dependent measures to quantify ANS acuity. *Accuracy* measures quantify ANS acuity as the total proportion of correct responses irrespective of the difficulty of the discrimination/detection task. The second type is *ratio* measures, which quantify ANS acuity as proportion correct or response time (RT) with respect to the ratio between the numeric stimuli. Finally, *internal weber fractions* model ANS acuity by estimating an individual *w*. The procedure of modeling ANS acuity is discussed further below.

#### Validity and reliability of ANS measures

While a lot of research has emphasized the relationship between ANS acuity and a variety of cognitive abilities (Halberda and Feigenson, 2008; Halberda et al., 2008; Mazzocco et al., 2011a,b) and cognitive impairments (Wilson et al., 2006; Räsänen et al., 2009; Mazzocco et al., 2011a,b) little attention has, until recently, been given to the reliability and validity of the tasks used to measure ANS acuity. Perhaps a bit surprisingly, recent research has raised concerns about both the reliability and validity of the tasks used to measure ANS acuity (Maloney et al., 2010; Gebuis and van der Smagt, 2011; Gilmore et al., 2011; Inglis et al., 2011; Price et al., 2012) thereby questioning at least some of the conclusions presented in previous work.

The results are mixed and while some studies report acceptable reliabilities (Gilmore et al., 2011; DeWind and Brannon, 2012; Halberda et al., 2012) others report moderate or low reliabilities (Maloney et al., 2010; Price et al., 2012). The inconsistencies in results might be attributed to, at least, three differences in methodology. First, there are several tasks that may be used to measure ANS acuity and studies reporting high or acceptable reliabilities have commonly used direct measures of ANS acuity (Gilmore et al., 2011; Halberda et al., 2012) while those reporting lower reliabilities have used indirect measures (Maloney et al., 2010). Second, the use of numeric distance as dependent measures seems to give lower reliability than the use of measures of internal *w*:s for some tasks. For example, Price et al. (2012) reported lower reliability for a numeric distance (ratio effect) measure than for *w* when the task presented stimuli intermixed while the reverse was true when stimuli was presented paired. Finally, the number of trials varies a lot over studies with some studies reporting reliabilities for several thousand trials (DeWind and Brannon, 2012); others report reliabilities for only a few hundred trials (Gilmore et al., 2011; Halberda et al., 2012). The classical true score model (Spearman, 1907) is based on the premise that test scores are fallible measures of human traits (true values). The reliability coefficient is defined as the ratio of true score variance to the total variance of test scores. In this model, reliability increases monotonically as a function of test length, following the law of diminishing returns, derived in the Spearman–Brown prophecy formula (Crocker and Algina, 1986). Because the reliability of a measure sets an upper bound on the correlation with other measures and because researchers often focus on relating ANS acuity to other measures of cognitive ability it is important to further investigate the reliability of ANS measures. In the present study we investigate two aspects of the reliability of ANS measures. First, we investigate reliability as a function of the number of trials in a discrimination task with simultaneously presented stimuli. Second, we investigate the upper bounds on the theoretical reliability.

### Modeling ANS acuity

The representation of magnitudes in the ANS is considered to be noisy in the sense that the representation of a specific numerosity varies. For example, the representation of the numerosity six is a random variable with a mean of six and a normally distributed variance. Further, representations become increasingly imprecise as numerosity increases. To model these representations two main approaches have been used, in both of which magnitudes are considered Gaussian random variables with mean equal to the actual numerosity. In the *logarithmic model* the means increase logarithmically with numerosity while the standard deviation is constant for all numerosities. In contrast, in the *linear model* both means and standard deviations increase linearly with numerosity. Even though these models make different assumptions about how the ANS represents magnitude they, in all but a few cases (Dehaene, 2003), make the same predictions regarding the ability to discriminate between magnitudes. Recently, several researchers (Halberda et al., 2008; Inglis et al., 2011; Lyons and Beilock, 2011; DeWind and Brannon, 2012; Price et al., 2012; Lindskog et al., 2013a) have used a classical psychophysics model suggested by Barth et al. (2006); see also, Pica et al. (2004), that relies on the linear model of the ANS, to model performance in ANS acuity tasks.

Given the linear model of the ANS, an optimal response strategy in a comparison task is to respond that the set associated with the larger internal representation is the more numerous (i.e., respond *n*_2_ > *n*_1_ whenever *n*_2_ – *n*_1_ > 0). Thus, with this model an optimal response strategy and the corresponding percentage of correct discriminations between two stimuli can be modeled as a function of the increasing ratio between the two sets [(larger sample (*n*_2_)/smaller sample (*n*_1_)]. The two sets are represented as Gaussian random variables with means *n*_1_ and *n*_2_ and standard deviations *w* · *n*_1_ and *w* · *n*_2_, respectively. The response criterion is found by subtracting the Gaussian for the smaller set from that for the larger, which gives a new Gaussian with a mean of *n*_2_−*n*_1_ and a standard deviation of $w\sqrt{n_{1}^{2}+n_{2}^{2}}$. The error rate in the comparison task is then the area under the tail (i.e., to the left of 0) of this resulting Gaussian and the proportion of correct responses can be computed as,

(1) $$1-\frac{1}{2}\text{erfc​}\left(\frac{\left|n_{1}-n_{2}\right|}{\sqrt{2}w\sqrt{n_{1}^{2}+n_{2}^{2}}}\right)\text{​},$$

where the term being subtracted is the error rate and erfc is the complementary error function. Equation 1 is fitted to the percentage correct discriminations as a function of the Gaussian approximate number representation for the two sets of stimuli with *w* as a free parameter. The *w* obtained describes the standard deviations of the Gaussian representations (i.e., how much the two Gaussian representations overlap) thereby predicting an individual's percentage correct on a discrimination task.

Even though the model described above has been frequently used, its performance with different number of observations has not been evaluated. In the present study, we present simulations that evaluate the performance of the model with respect to reliability. Previous research has indeed indicated the potential volatility of *w*, when fitting the model to a small number of data points (Mazzocco et al., 2011a,b; Odic et al., 2012).

### The present studies

In Study 1, we investigate the empirically observed and the theoretical maximum upper bound of reliability, as a function of the number of trials, for one of the standard tasks used to measure ANS acuity (Halberda et al., 2008). The former is estimated by reanalyzing previously collected data (Lindskog et al., 2013a). The results indicate that even in an ideal situation, large unreliability is introduced by an unavoidable binominal sampling error. Because of this sampling error, several hundred trials are needed to achieve an acceptable reliability. Therefore, in Study 2 we create a measure of ANS acuity requiring fewer trials to achieve acceptable reliability by introducing an adaptive test based on the ZEST algorithm (King-Smith et al., 1994). This adaptive test and other more conventional tasks are evaluated for their reliability and convergent/predictive validity.

## Study 1: empirical and theoretical reliability

In Study 1, we investigated three important questions with respect to the reliability of ANS-measures and models. First, we evaluated the empirical reliability for a standard task that measures ANS acuity, as a function of the number of trials. Second, using computer simulation we evaluated the theoretical maximum reliability as a function of the number of trials. Finally, we investigate if small samples introduce biases in the measures of *w*.

### Empirically observed reliability

To investigate the empirical reliability in a task that measures ANS acuity we used raw data from a study investigating rapid effects of feedback on ANS acuity (Lindskog et al., 2013a). In this study 39 participants (university undergraduate students, 31 female, with a mean age of 25.4 years, *SD* = 5.7) performed 1300 trials on a task that closely models the task used in Halberda et al. (2008). On each trial of the task, participants saw spatially intermixed blue and yellow dots on a computer monitor. The exposure time (200 ms) was too short for the dots to be serially counted. One of five ratios was used between the arrays (1:2, 3:4, 5:6, 7:8, 9:10) and the total number of dots varied between 11 and 30. One 5th of the trials consisted of each ratio. Half of the trials had blue and half had yellow as the more numerous set. The dots varied randomly in size. To counteract the use of perceptual cues dot arrays were matched either for total area or for average dot-size. The participants judged which set was more numerous by pressing a color-coded keyboard button.

Studies of ANS acuity have used test lengths that vary from 60 to 80 trials (Halberda et al., 2008; Libertus et al., 2011, 2012) up to several thousands of trials (DeWind and Brannon, 2012). It is therefore important to evaluate the reliability of ANS acuity tasks as a function of the number of trials. There are several procedures that could be used to estimate the reliability of a test. However, because *w* is used as a measure of performance procedures that estimate reliability using item covariance (e.g., Cronbach's alpha and KR 20; Crocker and Algina, 1986) cannot be used. We, therefore, estimated reliability by splitting the test into two halves and correlating performance on the two halves (split-half reliability; Crocker and Algina, 1986)^2^. By varying the number of trials in the full test we can thereby evaluate reliability as a function of the number of trials.^3^

The reliability of the task described above was estimated as a function of the number of trials (*N*) in the following way. For each *N* we estimated 100 reliability coefficients by randomly drawing two independent sets of data points for each participant and calculating the correlation between the two sets, both using the best fitting *w* and the observed proportion correct (*P*). The two sets of data points were drawn without replacement from the 1300 data points of each participant with the constraint that one fifth of the data points were required to come from each of the five ratios. We varied *N* in steps of 50 from 50 to 650^4^.

**Figure 1:**
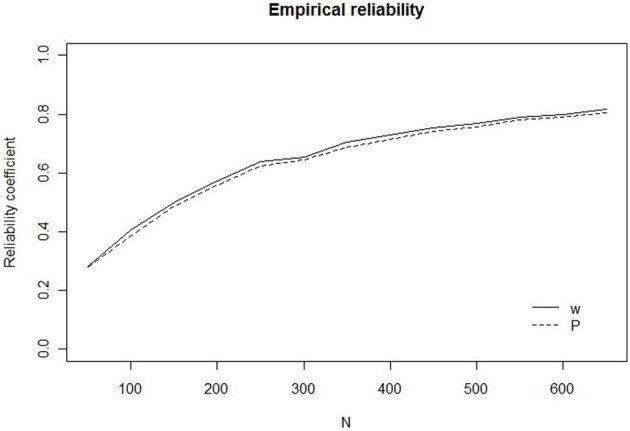
**Empirical reliability for *w* (solid line) and *P* (dashed line) as a function of the number of trials (*N*)**.

The results are illustrated in Figure 1 that shows reliability as a function of the number of trials, for *w* and *P*, respectively. The figure invites three major conclusions. First, the reliability of the task at small *N* (50–200), which are commonly used to measure ANS acuity, is low with a reliability coefficient below 0.5 for both *w* and *P*. It is noteworthy that *w*, in spite of being estimated through modeling stands up quite well-compared to *P* in terms of reliability. Second, for both measures it is necessary to have tests with about 400 trials to reach an acceptable reliability of 0.7. Finally, the reliability of the two measures is more or less identical for all *N*^5^. The original data only allowed us to estimate the exact reliability for *N* up to 650. Calculation of reliabilities corrected for test length, using the Spearman–Brown prophecy (Crocker and Algina, 1986), however, reveals that the rate with which the reliabilities increase quickly levels out when *N* becomes larger than 700. Increasing *N* from 700 to 1300 only increases the reliability from 0.81 to 0.89.

### The theoretical maximum reliability

The discrimination process is inherently stochastic; the same stimulus may lead to different responses from trial to trial. The proportions of correct responses (*p*) predicted by the model in Equation 1 are therefore expected values of the observed proportions of correct responses (*P*) found in empirical data. Because the process is stochastic *P* as a measurement of *p* will include an unavoidable sampling error. Further, because *P* comes from a binomial sampling process, the size of the sampling error will depend on the size (*N)* of the samples (i.e., the number of trials). For an analogy, consider when you measure the probability (*p)* that a certain tossed coin comes up heads (a stochastic process) by calculating the proportion of heads (*P)* in a sample of coin tosses. If you estimate *p* by tossing the coin 5 times, the sampling error of the binomial process with *N* = 5 will provide an upper boundary on how accurately you can estimate *p*. If you perform this “measurement” (i.e., use *P* as an estimate of *p*) repeatedly, each time tossing the coin 5 times, you will end up with different estimates of *p* each time and the variability of the estimates will be a function of the number of tosses. In other words, the sample size *N per se* defines an upper ceiling on the reliability in the measurement of *p*.

The sampling error that will always lead to imperfect reliability at small *N* has probably been of a non-trivial size in previous studies. For example, Libertus et al. (2012) (Experiment 1) fit *w* to ten stimulus difficulty ratios over a total of 60 trials, leaving six observations per ratio. Odic et al. (2012) concluded that it was not feasible to fit *w* reliably with seven difficulty ratios and 35 observations (five observations per ratio). Mazzocco et al. (2011b) reached the same conclusion when trying to fit *w* with 64 trials consisting of nine ratios, of which four were estimated with two observations each. Thus, it is not uncommon that researchers have attempted to measure *w* with few observations. In the following, we describe a simulation, which estimates the theoretical upper limit for the reliability coefficient, where we use the linear model (Equation 1) to produce an individual *w* as a measure of performance.

We relied on a distribution of 224 empirically observed *w-values* from adult undergraduate students tested in our lab. The *w*-values had a median of 0.22 and an inter-quartile range of 0.09, similar to previous published research (Pica et al., 2004; Halberda and Feigenson, 2008; Halberda et al., 2008; Tokita and Ishiguchi, 2010). We simulated 100 “experiments,” with 40 “ideal participants” each responding to the same five stimulus ratios as in the above analysis of the empirical reliability. The “participants” are ideal in the sense that the responses were computed from Equation 1, with the only origin of unreliability therefore deriving strictly from the binomial sampling error. The data were thus generated from Equation 1 and a binomial sampling process, for sample sizes *N* in steps of 50 from 50 to 650. Specifically, in each “experiment,” we sampled a random set of 40 *w*-values from the distribution described above. We generated two data sets, *A*_*i*_ and *B*_*i*_, for each “participant” (*i* = 1 … 40) in each “experiment,” by first calculating the expected value *p*_*ij*_ of the proportion correct for each of the five ratios *j* given an true value of *w*_*i*_ (Equation 1). We then generated the two parallel sets of data points from a binomial distribution with *n* = *N*/5 and *p* = *p*_*ij*_. For the two sets of *N* data points, we thereafter calculated the overall proportion correct *P*_*Ai*_ and *P*_*Bi*_ and the best fitting estimates, *W*_*Ai*_ and *W*_*Bi*_, of *w*_*i*_, respectively. The reliabilities of *P* and *W* were obtained by calculating the correlation between the two data sets for each measure. Because the irreducible sampling error is the only source of unreliability in the simulations, these coefficients represent the highest reliability that is possible at sample size *N* according to the underlying model.

**Figure 2:**
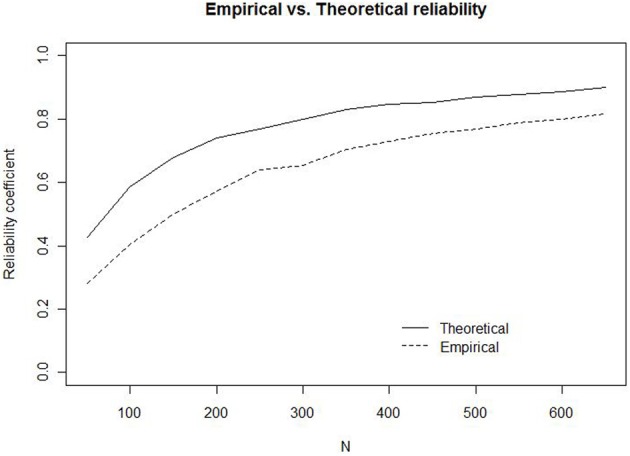
**Empirical (dashed line) and Theoretical (solid line) reliability for *w* as a function of the number of trials (*N*)**.

The simulations for *w* are illustrated in Figure 2 (solid line) together with the results from the estimation of the empirical reliability (dashed line)^6^. The results invite two major conclusions. First, the theoretical maximum reliability is higher than the empirical reliability for all *N*, because the simulations ignore the additional unreliability introduced by the use of human participants. Second, at small *N* (50–200), the sampling error alone is sufficient to produce a theoretical maximum reliability that is rather low (0.42–0.74). For example, with 50 trials, corresponding to 10 observations per ratio, the highest possible reliability in this setting is 0.42. To have a theoretical maximum reliability of 0.8, more than 300 trials are required. To estimate the attenuation of reliability from the additional variability introduced by human participants, we calculated the differences between the theoretical and the empirical reliabilities. For experiments with fewer than 600 trials, we expect to find reliabilities that are at least 0.1 lower than the theoretical maximum reliabilities displayed in Figure 2,^7^.

A direct implication of this analysis is that the sampling error (i.e., [*p*·(1–*p*)]/*N*) is larger for difficult stimuli (*p* close to 0.5) than for easy stimuli (*p* close to 1)^8^. To illustrate this, consider the case where *N* is 240 and all five stimulus ratios are equally present, as in the above analysis, where the reliability is 0.77. If only the two easiest ratios (1:2, 3:4) are used the reliability at *N* = 240 is 0.88, but if only the most difficult ratios (8:9, 9:10) are used the reliability is 0.64^9^. The simulation also allows us to evaluate if the linear model introduces biases into the estimates of *w* at small sample size; if the model fitting procedure provides estimates *W* of *w* that are systematically larger or smaller than the true population value of *w*. We therefore calculated the mean difference between the *w* used to generate the data and the fitted *W* for each *N*. This analysis revealed that the mean difference did not deviate significantly from 0 for any *N*. We also looked at the possibility that using easy/hard subsets of stimuli would bias the estimates of the population *w*, but there were no signs of this. That is, the modeling procedure does not introduce systematic biases into estimates of *w*.

### Discussion

In Study 1 we estimated the empirical reliability of a task, used by several researchers, that measures ANS acuity. The results indicated poor reliability, which was similar for both *w* and proportion correct, with the number of trials (50–300) most commonly used. The results indicated that more than 600 trials were needed to reach an acceptable reliability of 0.8. The low reliability may at least in part derive directly from the binomial sampling error that is an unavoidable consequence of measuring discrimination performance with proportions.

To estimate the attenuation of reliability introduced by sampling error, and to evaluate the linear model, Study 1 used computer simulation to evaluate the theoretical reliability as a function of number of trials. Even in ideal circumstances it was not possible to reach acceptable reliability at low *N*. Approximately 350 trials were needed to reach a reliability of 0.8 and an additional 1000 trials were required for a reliability of 0.95. Even if it would be possible to eliminate all of the errors due to human factors from the measurements, sampling error alone will attenuate reliability. The analysis also showed that tests made up of easier stimuli are more reliable and that the linear model provides unbiased estimates of *w*.

Although the reliability functions in Figure 1 are far from universal, and will be affected by, for example the stimuli of a particular study, it is interesting to make comparisons to estimated reliabilities in previously published papers. With this respect a few estimates stick out (e.g., Gilmore et al., 2011) with reliabilities that seem higher than possible even with error free measurements. A possible explanation of such “too high” observations is probably that measurements have been polluted, assessing something else above the ANS. This will lead to correlated error measurements that violate an assumption of classical test theory (Crocker and Algina, 1986, p. 114). A plausible candidate for a variable that may affect results is the influence of perceptual variables on behavior. In general, in numerosity judgment tasks, stimuli are arranged in such a way as to control for use of perceptual variables (e.g., cumulative area). This set up leads to a situation in which those who rely on perceptual variables will take a hit in performance measures. Because those who rely on such cues will probably do so throughout the experiment, this will lead to correlated errors of measurement when for example calculating split-half reliability, giving an inflated false impression of high reliability. Another arrangement that may lead to correlated errors of measurement is the increasing use of data collected on the internet (e.g., Halberda et al., 2012). While such data collections make it possible to effortlessly obtain very large samples of participants they have the drawback that the researcher has limited or no control of conditions during stimulus presentation. Thus, if one individual takes the test under poor viewing conditions, this will most probably affect performance detrimentally on both test halves, again leading to a false sense of reliability in measures. Thus, extremely high reliability estimates may not be entirely positive, but signal that the test measures other variables than the ANS.

To conclude: the presence of irreducible sampling error implies that we need a large number of trials to reach good reliability with the procedures and models commonly used in research. Easier stimuli are associated with more reliable measures than harder stimuli.

## Study 2: an adaptive measure of ANS acuity

The results from the simulations highlight that the traditional measures of ANS acuity might suffer from low reliability, at least for small *N*. It is of course possible to have participants do a very large number of trials in order to achieve a reliable measure of ANS acuity. However, such procedures quickly become time consuming for the researcher and tedious for the participant, which in turn might introduce more noise into measurements. It would be preferable to have a task that can produce reliable measures of ANS acuity with as few trials as possible. While sampling error *per se* cannot be eliminated, one can select the most diagnostic stimulus on each trial with an adaptive test procedure. We developed and evaluated an adaptive measure based on the ZEST-algorithm (King-Smith et al., 1994) in Study 2. The results from the Study 1 also indicated that because reliability is an upper bound on the observable correlation between two measures, the previously reported correlations might have been attenuated by low reliability. To evaluate this possibility, Study 2 included measures of arithmetic fluency, intelligence and perceptual speed. With a more reliable ANS acuity measure based on the ZEST-algorithm we hoped to gain new insight into the predictive validity issue in terms of concurrent correlations with arithmetic fluency. Finally, the fact that different studies use different methods makes comparisons between measures difficult and renders within-subjects studies valuable.

### Methods

#### Participants

Participants (13 Male, 27 Female) were undergraduate students from Uppsala University with a mean age of 24.6 years (*SD* = 8.2 years). They received a movie ticket or course credits for their participation.

#### Materials and procedure

Participants carried out a set of six tasks, described in detail below, developed to measure ANS acuity, perceptual processing speed, intelligence, and arithmetic fluency.

***Static non-symbolic number comparison.*** The static non-symbolic number comparison task was based on Halberda et al. (2008). On each of the 100 trials, participants saw spatially intermixed blue and yellow dots on a monitor. Exposure time (300 ms) was too short for the dots to be serially counted. We used five ratios between the two sets of dots (1:2, 3:4, 5:6, 7:8, 9:10) with the total number of dots varying between 11 and 30. One fifth of the trials consisted of each ratio. Half of the trials had blue and half had yellow as the more numerous set. The dots varied randomly in size. To counteract the use of perceptual cues we matched dot arrays either for total area or for average dot-size. The participants judged which set was more numerous by pressing a color-coded keyboard button.

***Adaptive non-symbolic number comparison.*** The stimuli were the same type of dots and had the same color as those in the static test. ANS threshold for obtaining 80% correct discriminations was estimated by an adaptive method, the ZEST algorithm (a modification of the Bayesian QUEST algorithm, King-Smith et al., 1994). The algorithm calculates the stimulus difference for each trial based on the performance on earlier trials in the discrimination task. Weber fractions *w*—ΔS/S, where *S* is a stimulus parameter (number of dots) and ΔS is the interstimulus difference—were used to quantify the difference between stimulus pairs at each trial. The ZEST algorithm uses all responses in previous trials for optimal estimation of the difference between stimuli presented in the next trial and converges to the threshold estimate, *w*, for achieving the desired percentage of correct responses. In short, after each trial this method multiplies a probability density function (a prior PDF) of *w* with a likelihood function of obtaining the response (correct or incorrect). The result is an updated density function (a posterior PDF). The mean of the updated PDF is used to determine *w* of the next trial. A loop was used that searched for the nearest *w* ratio with integer composition of dots with the constraint that the total number of dots not exceeded 33, and a minimum of six dots of the less numerous set. If several ratios were found, the ratio with lowest total number of dots was used. This procedure was repeated in a predetermined number of trials and the final *w* was used as the threshold estimate. The initial PDF was a normal distribution of possible *w*:s with an average of 0.23 and standard deviation 0.58. The initial estimate of *w* was based on the median *w* for a large number of participants (~200) tested in our lab. This estimate is also consistent with a large number of previous studies (e.g., Pica et al., 2004; Halberda and Feigenson, 2008; Halberda et al., 2008; Tokita and Ishiguchi, 2012). Participants received 240 trials, for which the algorithm simultaneously estimated two *w-*values based on randomly ordered intermixed trials (120 trials each). We used both a simultaneous and a sequential version. In the sequential version, the two numerosities were separated by an ISI of 300 ms with a blank screen.

***Symbolic numeric distance effect.*** We used a symbolic number comparison task based on Moyer and Landauer (1967). On each of the 160 trials participants saw a fixation cross during 500 ms followed by two Arabic digits positioned to the left and right of the fixation cross. Each trial presented a *standard* digit, which was always the digit 5, with a *comparison* digit, which was either smaller (1, 4) or larger (6, 9) than the standard digit. Thus, the comparison digit was either close to (4, 6) or far from (1, 9) the standard digit. The digits remained visible until participants had responded. The task was to decide if the comparison digit was smaller or larger than the standard digit and we measured the response time (RT) from the presentation of the digits until the response was given. Standard and comparison digits were randomly assigned to be presented to the left or right of the fixation cross for each trial. The symbolic NDE was calculated as the dependent measure. Following Holloway and Ansari (2009) and Gilmore et al. (2011) we defined the NDE as (*RT*_*C*_—*RT*_*F*_)/*RT*_*C*_ where *RT*_*F*_ and *RT*_*C*_ are mean RT on trials were numbers differed more and trials where numbers were closer in numerosity, respectively^10^.

***Non-symbolic numeric distance effect.*** Two measures of non-symbolic distance effect were used based on the responses of the static non-symbolic number comparison task described above^11^. The first was defined as (*RT*_9:10_—*RT*_1:2_)/*RT*_1:2_ where *RT*_1:2_ and *RT*_9:10_ are mean RT for trials with ratio 1:2 and 9:10, respectively. We defined the second measure analogously as (*P*_9:10_—*P*_1:2_)/*P*_1:2_, where *P* is proportion correct.

Before calculating the two NDE measures based on RT, individual responses were scanned for outliers and responses with an RT of more than three standard deviations were excluded. This procedure excluded ~2% of individual responses.

***Perceptual speed.*** The “visual inspection time” task, that measure general perceptual and mental speed, was closely based on Deary et al. (2004). On each trial participants saw a fixation cross for a duration of 500 ms, followed by a stimulus with one horizontal line and two vertical lines organized to resemble the Greek letter Π. The two horizontal lines were of different lengths and participants had to decide which of the two was the longest. The Π-stimulus was presented with one of five presentation times (25, 40, 60, 80, and 100 ms) and participants saw 20 presentations from each presentation time. The Π-stimulus presentation was followed by a 500 ms mask covering the two horizontal lines. Participants gave their answer by pressing the letter *F* (left was longer) or the letter *K* (right was longer) on a computer key board.

***Raven's matrices.*** Participants carried out a subset of Raven's progressive matrices (Raven et al., 1998) based on Stanovich and West (1998) (see also Carpenter et al., 1990). Participants were first instructed on the task. They were then allowed two of the 12 test items before completing 18 of the test items (item 13 through 30) with a 15 min time limit. Participants were instructed to try to complete all 18 items within the time limit.

***Arithmetic fluency.*** The arithmetic fluency task was based on the mathematical task found in Gebuis and van der Smagt (2011) and consisted of four sets of arithmetic problems; *addition, subtraction, multiplication,* and *division*. For each set participants had 150 s to complete as many problems as possible. Each set presented problems with increasing difficulty accomplished by adding more digits and requiring borrowing or carrying. For example the first three problems in the addition and multiplication sets were 2 + 7, 12 + 9, and 38 + 17, and 2· 3, 3 · 6, and 4 · 7, respectively. The order of sets was counterbalanced over participants.

### Results and discussion

For the adaptive ANS acuity tasks the dependent measure was the posterior estimated individual *w* score. For the non-adaptive ANS acuity task we calculated an individual *w* score and mean proportion correct (*P*). In the other tasks we used proportion correct (inspection time task) or number of correct answers (arithmetic fluency task, Raven's matrices) as dependent measure. All dependent measures were scanned for outliers using z > |3.5|, which led to no data points being excluded.

#### Reliability

Participants performed two rounds of the inspection time task, the symbolic number comparison task and the two versions of the adaptive non-symbolic number comparison task. To evaluate the reliability of these four tasks we calculated the correlation between the first and the second round. The results are summarized in Table 1, which also includes full length reliability coefficients corrected for test length using the Spearman–Brown prophecy formula. The results indicate that while reliability is acceptable for the sequential adaptive task already at test length of 120 trials, the simultaneous adaptive task and the Symbolic distance effect require at least 240 and 320 trials, respectively, to at least approach acceptable levels of reliability. There is no obvious difference between direct and indirect measures of ANS acuity with respect to reliability. The non-symbolic NDE based on *P* entirely lacks reliability. The corrected reliability for the perceptual speed task (not included in the table) was 0.77, corrected for a test length of 320 trials.

**Table 1:** **Reliability coefficients and Spearman–Brown corrected reliability coefficients for the different proposed measures of ANS-ability**.

**Measure**	**Uncorrected**	**Corrected**
**DIRECT**		
Static^a^		
*w*	0.40 (100)	n.a.
*P*	0.40 (100)	n.a.
Adaptive		
Simultaneous	0.58 (120)	0.74 (240)
Sequential	0.85 (120)	0.92 (240)
**INDIRECT**		
Distance effect		
Symbolic	0.53 (160)	0.69 (320)
Non-symbolic^a^		
RT	0.39 (100)	n.a.
*P*	0.15 (100)	n.a.

Note: The direct test measures were the dependent measures obtained with the static task based on Halberda et al. (2008) and the simultaneous and sequential adaptive task. The indirect test measures were the symbolic and non-symbolic distance effects. The numbers in brackets after the reliabilities indicate the number of trials for which the reliability was estimated.

a The reliability of the measures are based on the evaluation of empirical reliability from Study 1.

#### Convergent validity of ANS acuity measures

We investigated the convergent validity of the different measures of ANS acuity (i.e., the extent to which they measure the same construct) by calculating all pairwise correlations between the measures of ANS acuity. For the tasks that participants performed twice we constructed an aggregated score before calculating the correlations. Table 2, which includes correlations adjusted for reliability, displays the results^12^. As can be seen, there are several significant correlations. However, considering that all measures are expected to measure the same construct correlations are moderate to low, with the exception of the both dependent measures of the static tasks (*w, P*) (*r* = 0.95). These low correlations could, be the result of low reliability. Correcting for reliability reveals that there are, indeed, strong relationships between some of the measures. For example, the corrected correlations between the static and adaptive simultaneous tasks (*w*) (*r* = 0.88), (*P*) (*r* = 0.88), are very high, as well as the correlation between the two dependent measures calculated for the static direct task (*r* = 0.95). The adjusted correlations between the non-symbolic NDE measure for *P* and the static task measures measure are also high. (However, all adjusted measures for the non-symbolic *P* effect should be interpreted with caution, since the extremely low reliability for this variable will boost all correlations greatly.) A bit surprisingly, in spite of the high reliability found for the sequential discrimination task *w*-measure, this variable is unrelated to all other measures, even after adjustment for attenuation. The most striking result is that the non-symbolic (RT) NDE measure correlates negatively with all direct task measures. The adjusted negative correlation between this measure and the adaptive simultaneous task is even extremely high (−0.89). While measures 1–4 are direct measures of ANS acuity measures 5–7 are indirect measures. The results indicate that there is a weak or negative relationship between these two types of measures. The only positive correlation between these measures is the correlation between the symbolic distance task and the adaptive simultaneous task (0.52 adjusted). However, the results for this variable are inconclusive, since no correlation was found for the other direct measures. It should also be noted that performance on the perceptual speed task (not in the table) correlated significantly only with the symbolic version of the NDE [*r* = 0.38 (0.45)]^13^.

**Table 2:** **Pairwise correlations between proposed measures of ANS capacity**.

	**Task (Measure)**						
**Task (Measure)**	**1**	**2**	**3**	**4**	**5**	**6**	**7**
**ADAPTIVE**							
1. Simultaneous	**0.74**						
2. Sequential	0.34^*^ (0.41)	**0.92**					
**STATIC**							
3. (*w*)	0.48^*^ (0.88)	0.00 (0.00)	**0.40**				
4. (*P*)	0.48^*^ (0.88)	−0.03 (−0.05)	0.95^*^^a^	**0.40**			
**NUMERIC DISTANCE**							
5. Non-symbolic (RT)	−0.48^*^ (−0.89)	−0.11 (−0.18)	−0.41^*^^a^	−0.42^*^^a^	**0.39**		
6. Non-symbolic (*P*)	0.14 (0.42)	−0.15 (−0.40)	0.23 (0.94)	0.41^*^^a^	−0.04 (−0.17)	**0.15**	
7. Symbolic	0.37^*^ (0.52)	0.17 (0.21)	−0.04 (−0.08)	0.01 (0.02)	−0.11 (−0.21)	−0.08 (−0.25)	**0.69**

Note: Correlations in bold along the diagonal are reliability coefficients for the current test length. Signs of the correlations have been transformed so that a positive sign indicates a positive functional association. Included in brackets are correlations adjusted for reliability.

* p < 0.05.

a Corrected reliabilities exceeded 1 and were therefore excluded.

The results of the analyses of convergent validity indicate that all of the measures that have been used in previous research do not measure the same construct. More specifically, there seems to be a weak or even negative relationship between direct and indirect measures of ANS acuity, suggesting that some of the mixed results reported in previous work (Halberda et al., 2008; Inglis et al., 2011) might very well be due to methodological differences.

#### Predictive validity^14^

The predictive validity of the tasks used to measure ANS acuity was evaluated by calculating the Pearson correlation between the measures and all four subtests of the arithmetic fluency task, an aggregated measure of arithmetic fluency and performance on Raven's matrices. The results, summarized in Table 3, show that while the direct measures that use simultaneous presentation are related to arithmetic fluency, the sequential adaptive measure and the indirect measures are not. For the non-symbolic (RT) measure, the sign of all correlations is negative, even though not statistically significant. Notice that it is foremost a somewhat simpler, or basic, arithmetic fluency (BARF) in terms of an addition and subtraction skill that is related to ANS acuity, rather than the more advanced skills of multiplication and division. Division performance does not correlate with any measure. Further, none of the seven measures are significantly related to performance on Raven's matrices.

**Table 3:** **Correlations between all measures of ANS acuity and arithmetic fluency and intelligence (Raven's matrices)**.

	**Math task**					
**Acuity task**	**Addition**	**Subtraction**	**Multiplication**	**Division**	**Total**	**Raven's**
**ADAPTIVE**						
Simultaneous	0.43^*^	0.31^*^	0.30^**^	0.10	0.32^*^	0.22
Sequential	0.07	0.12	0.02	−0.14	0.02	0.22
**STATIC**						
(*w*)	0.32^*^	0.39^*^	0.20	0.07	0.28^***^	0.09
(*P*)	0.40^*^	0.41^*^	0.28^***^	0.16	0.35^*^	0.09
**NUMERIC DISTANCE**						
Non-symbolic (RT)	−0.28	−0.24	−0.12	−0.11	−0.22	−0.15
Non-symbolic (*P*)	0.11	−06	0.03	0.13	0.10	−0.12
Symbolic	0.23	0.07	0.18	0.09	0.16	0.01

Note: Signs of the correlations have been transformed so that a positive sign indicates a positive association.

* p < 0.05,

** p = 0.06,

*** p = 0.08.

To investigate the possibility that basic arithmetic fluency (BARF) could be predicted by ANS acuity after controlling for intelligence and perceptual speed we ran multiple regression analyses for each proposed measure of ANS acuity, with a composite measure of performance on addition and subtraction as dependent variable and controlling for perceptual speed, and intelligence (Raven's matrices). The results, summarized in Table 4, indicate that the three measures obtained by direct simultaneous tests predict performance in basic arithmetic tasks even after controlling for perceptual speed and intelligence. No other measures of ANS are even close of being significantly related to arithmetic fluency.

**Table 4:** **Multiple regression models for each ANS acuity measure, with basic arithmetic fluency (addition and subtraction) as dependent variable and perceptual speed, intelligence (Raven's matrices) as predictors**.

**Acuity task**	**ANS**	**Speed**	**Raven's**	***R***^**2**^ **(model *p*)**
**ADAPTIVE**				
Simultaneous	0.33^*^	0.00	0.28^***^	0.23 (0.02)
Sequential	0.02	0.01	0.35^*^	0.13 (0.18)
**STATIC**				
(*w*)	0.36^*^	0.08	0.31^*^	0.25 (0.01)
(*P*)	0.40^*^	0.07	0.30^*^	0.29 (0.01)
**NUMERIC DISTANCE**				
Non-symbolic (RT)	−0.23	0.03	0.31^**^	0.18 (0.07)
Non-symbolic (*P*)	0.14	0.01	0.37^*^	0.14 (0.13)
Symbolic	0.17	0.04	0.36^*^	0.15 (0.11)

Note: Signs of the beta-weights have been transformed so that a positive sign indicates a positive association.

* p < 0.05,

** p = 0.05,

*** p = 0.07.

#### Stimulus difficulty and predictive validity

The analysis of reliability in Study 1 revealed higher reliabilities for easier stimuli. We therefore performed corresponding analyses for predictive validity estimates for the different stimulus ratios of the static task (*P*). The zero order correlations between the test previously shown to be associated with highest reliability (made up of the 1:2 and 3:4) ratios with BARF was not significant [*r*_(38)_ = 0.21, *p* = 0.19]. The corresponding correlation for the test previously shown to be associated with lowest reliability (made up of the 7:8 and 9:10) ratios, however, was significant [*r*_(38)_ = 0.38, *p* = 0.017]. Thus, paradoxically, the stimuli with lower reliability showed predictive validity, but not the stimuli with higher reliability. We conducted a multiple regression analysis with performance at each of the stimuli ratios (*P*) as predictor variables and BARF as dependent variable. The results are shown in Table 5, which reveals that the 7:8 ratio is the only stimulus that contributes to predictive validity. Removing all other predictor variables lead to no significant reduction in *R*^2^. The easier stimuli (ratio 1:2) do even have negative beta-weights.

**Table 5:** **Multiple regression models [(beta-weights (*p*-value)], with arithmetic fluency as dependent variable and discrimination performance (*P*) at the different stimulus ratios (difficulties) as predictors**.

**Dependent**	**Predictor**					
	**1:2**	**3:4**	**5:6**	**7:8**	**9:10**	***R***^**2**^
Basic	−0.12 (0.41)	0.14 (0.38)	0.26 (0.11)	0.41 (0.008)	0.11 (0.47)	0.28
Advanced	−0.30 (0.06)	0.036 (0.82)	0.23 (0.18)	0.34 (0.033)	0.04 (0.81)	0.23
Total	−0.21 (0.16)	0.09 (0.56)	0.25 (0.12)	0.39 (0.012)	0.08 (0.61)	0.28

#### Gain in reliability by the adaptive test

The results in Tables 3, 4 indicate that both the simultaneous direct discrimination tasks have significant predictive validity. However, as can be seen in Table 1 there is a considerable difference in reliability for the same number of trials. To estimate the gain in reliability of using the adaptive simultaneous over the static task we used the Spearman–Brown prophecy to estimate the number of trials required to achieve an acceptable (0.8), high (0.9), and very high (0.95) reliability for the two tasks. The results are summarized in Table 6 and indicate that the adaptive task requires about 40% fewer trials than the static task to reach the same level of reliability. Thus, using ~350 trials the adaptive task is a measure of ANS acuity, which is both highly reliable and valid.

**Table 6:** **The (approximate) minimum number of trials needed to reach a reliability of 0.8, 0.9, and 0.95 for the adaptive and static simultaneous tasks, respectively**.

**Measure**	**Desired reliability**		
	**0.8**	**0.9**	**0.95**
Adaptive-Simultaneous	350	770	1630
Static (*w*/*P*)	600	1350	2900

## General discussion

When chasing after one of the groups of gazelles on your hunting expedition you can only hope that your ANS has helped you make a correct decision. Even though previous research has not investigated how individual differences in ANS acuity relate to decisions like the one on your hunting expedition *per se*, an extensive body of research has investigated how ANS acuity relates to a variety of cognitive abilities (Halberda and Feigenson, 2008; Halberda et al., 2008; Mazzocco et al., 2011a,b) and cognitive impairments (Wilson et al., 2006; Räsänen et al., 2009; Mazzocco et al., 2011a,b). However, the methods used to measure ANS acuity are many and until recently not much attention has been given to the reliability and validity of these methods (but see Maloney et al., 2010; Gebuis and van der Smagt, 2011; Gilmore et al., 2011; Inglis et al., 2011; Price et al., 2012). The large differences in tasks and dependent measures might be one explanation for the somewhat mixed results from previous research.

In the present paper we conducted two studies to further investigate issues concerning reliability and validity of ANS acuity measures and to, if possible, develop a task that measures ANS acuity reliably in a reasonable number of trials. In Study 1 we evaluated the empirical reliability of a task that is commonly used to measure ANS acuity as a function of the number of trials participants need to perform. The results indicated that a substantial number of trials were needed to reach an acceptable reliability and that previous studies using small numbers of trials probably suffer from low reliability. The results from the simulations showed that even in a world with perfect error free measurements, sampling error alone will attenuate the reliability coefficients when the number of trials is low. Analyses of stimulus difficulty and reliability revealed that easier stimuli prove more reliable than the hard, probably due to smaller error variance in these stimuli.

The results from Study 1 indicated the need for a task that can measure ANS acuity with acceptable reliability using a limited number of trials and without a requirement of *post-hoc* estimation of *w* using the linear model. The aim of Study 2 was to develop such a task based on the ZEST algorithm. Study 2 was also designed to further evaluate the reliability and validity of different measures of ANS acuity using a within-subject design.

The results of Study 2 revealed that the adaptive task was more reliable than the static task with the same number of trials. In fact the adaptive task could reach a given reliability in ~60% of the number of trials required for the non-adaptive task. Further, an analysis of convergent validity indicated that while direct measures with simultaneous stimulus presentation are highly related, indirect measures and sequential tasks are not. The non-symbolic (RT) NDE measure has been called a *“key-metric of ANS acuity”* (Price et al., 2012, p.50). Remarkably, this measure showed a statistically significant negative convergent validity with the direct measures. We know of only one other study (Price et al., 2012) that has calculated the NDE (RT) measure and *w* on the same data. Price and colleagues found no correlation between these measures on two out of three tests and a significant negative correlation on the third, a finding that together with our results suggest that indirect and direct indices do not measure the same thing. This is further supported by results from a recent article (Nys et al., 2013) showing that unschooled adults who had never received math education had both a larger error rate and a smaller NDE on a non-symbolic comparison task, than did schooled adults who had attended regular school in childhood. That is, poorer discriminability (i.e., larger *w*) was related to a smaller NDE.

The idea that the NDE can be used as an index of the quality of the representation of number has been very influential, but its origin is vague and not well-supported empirically. It probably comes from the attractiveness of theoretical models that by assuming a Gaussian internal number coding and sequential accumulation of evidence can generate relations between RT, accuracy and number distance that mimic the NDE (see Dehaene, 1992, 2007, 2009; Link, 1992; Cordes et al., 2001). In these models, the degree of overlap between representations (the imprecision of the representations) determines the size of the NDE (but see Van Opstal et al., 2008 for an account of number representation that gives the NDE without assuming overlapping representations). If future research confirms the suspicion that the non-symbolic NDE is not an appropriate index of the precision of non-symbolic number representations, previous research has to be reinterpreted. For example, Holloway and Ansari (2009) observed a correlation between the symbolic distance effect and mathematics achievement, but no corresponding correlation for a non-symbolic NDE. Mundy and Gilmore (2009) found the same pattern of results. However, their results showed that while the non-symbolic NDE did not correlate with math performance, overall performance on the non-symbolic task did. This result also suggests that the non-symbolic NDE is a poor estimator of non-symbolic representation precision. Holloway and Ansari (2009) suggested that their results questioned both the notion that the processing of non-symbolic magnitude serves as a precursor to the symbolic representation of number and that mapping of abstract symbols onto non-symbolic representations plays an important role when acquiring Arabic numerals. However, this finding could be accounted for by the non-symbolic NDE not being a valid measure of non-symbolic number representation precision. Thus, several studies, all using direct measures of the acuity of non-symbolic number representations suggest that there is such a connection (Halberda et al., 2008; Piazza et al., 2010; Libertus et al., 2011; Mazzocco et al., 2011a,b; Desoete et al., 2012).

Further, only the direct measures with parallel, simultaneous stimulus presentation exhibited predictive validity while indirect measures of ANS acuity were neither reliable nor showed predictive validity. The non-symbolic (RT) measure in the present study was not reliable, it lacked convergent validity, or showed even negative convergent validity, and the predictive validity coefficients were non-significant and of negative signs. All three dependent measures calculated from tests with parallel stimulus presentation on the other hand predicted arithmetic fluency even after controlling for general cognitive functioning and perceptual speed. These results are in contrast to the results of Price et al. (2012) who did not find correlations between arithmetic fluency and a direct test with parallel stimulus presentation. One possible explanation for this discrepancy is the use of different measures of mathematical achievement. While we use a simple measure of arithmetic fluency, Price and colleagues used the more complex Woodcock Johnson Math Fluency subtest (Woodcock et al., 2001). Another possibility is that the population correlation is low. If so, larger sample sizes would be required to increase the statistical power of documenting the relationship consistently.

In line with previous research (Price et al., 2012) we found no predictive validity for the sequential direct task. This is interesting, because the difference cannot be attributed to lack in reliability in the sequential measure, which was higher than in the direct tests with simultaneous stimulus presentation. The reason for this lack of predictive validity is unclear. One possibility is that sequential tests involve working memory processes in that the first numerosity needs to be retained in memory when comparing it to the second (as suggested by the factor analysis). It is also possible that the in psychophysical discrimination tasks commonly observed time-order error (TOE) plays a role in the sequential task. The TOE suggests that when two stimuli are presented sequentially, the second is experienced as more intense. We have (Lindskog et al., 2013b) shown that the TOE does occur for numerosity judgments. It is possible that in sequential tasks individual differences in *w* reflect differences in susceptibility to such TOE effects. This could, of course, only be the case if individual differences in TOE are not critical in arithmetic ability. Based on previous research there are at least no obvious a priori reasons which suggest that arithmetic ability should covary with TOE. Most studies on adults that have shown predictive validity have used parallel stimulus presentation, but such validity has also been shown in at least one study with sequential presentation (Gebuis and van der Smagt, 2011). It is unclear what could account for these differences, but a discrepancy to the present study, is that Gebuis and van Der Smagt used a longer inter-stimulus interval (800 ms) and a constant standard stimulus reappearing on all trials. Another interesting finding is that performance measures using proportion correct and *w* on direct tests showed virtually identical properties both for reliability and predictive validity. This means that the rather cumbersome fitting procedure involved in obtaining *w* estimates is unnecessary when absolute estimates of this measure is not the primary objective.

In order to measure ANS acuity with a measure that is both reliable and valid, it is preferable to use a direct measure with simultaneous stimulus presentation. Further, using the adaptive task developed in Study 2 this could be done more efficiently, with respect to the number of trials, than by using previously suggested methods (e.g., Halberda et al., 2008).

Whereas easier stimuli were more reliable, paradoxically these stimuli were found not to contribute to predictive validity, which suggests an intriguing trade-off between reliability and validity. Predictive validity was quite narrowly pinned down to the 7:8 ratio in the present study. This finding clearly needs to be replicated since it is based on very limited sample sizes. However, the finding suggests that it is only discriminations at a particular difficulty that are critical for obtaining correlations with arithmetic performance. This “optimal” difficulty level probably varies between participant populations, so that it for example is at a different level for children than for adults. This is, in addition to the fact that more difficult stimuli will demand more trials to reach an adequate reliability, another reason that speaks for the use of adaptive tests that adjust the difficulty level to an individual level. The result that predictive validity may be found only at an appropriate level of difficulty possibly also has contributed to the inconsistent results in previous research.

The growing interest in the ANS and how individual differences in the system relate to other cognitive abilities highlights the need for reliable and valid measures of ANS acuity. The present paper contributes with an analysis of commonly used measures and models. However, further research is needed to thoroughly map out how different tasks that measure ANS acuity are related and to which extent they measure what is intended.

### Conflict of interest statement

The authors declare that the research was conducted in the absence of any commercial or financial relationships that could be construed as a potential conflict of interest.

## Footnotes

^1^Previous research has used the terms *mathematical ability and mathematical achievement* broadly to include skills such as arithmetic ability and mathematical problem solving and even self-reported SAT-scores. In this study we are primarily interested in the more specific ability of *arithmetic fluency*. However, in the introduction we will use these terms interchangeably.

^2^While the choice of calculating split-half reliabilities over for example test-retest reliability allow us to investigate how errors in measurement affect reliability in a test situation on a single occasion it, of course, limits the possibility to evaluate how measurements fluctuate when they are separated by a given time period.

^3^It is, of course, possible to use the Spearman–Brown prophecy to estimate the reliability of different test lengths when the reliability of a given test length is known. However, this procedure only gives an approximation of the reliability while our procedure gives the exact reliability.

^4^In the study by Lindskog et al. (2013a) half of the participants received feedback on 1000 of the trials. This manipulation resulted in a small effect which disappeared when controlling for motivation. To assure that the manipulation did not affect the results of the simulations we reran all simulations, with almost identical results, with data from only the control group. To retain more reliable results, both groups are included in the analysis.

^5^Because some studies (e.g., Halberda et al., 2008) use four, rather than five ratios we reran all analyses using four ratios. The basic shape of the reliability-trial function was highly similar for both number of ratios but reliabilities were ~5% higher when using four as opposed to five ratios.

^6^The results were similar for proportion correct.

^7^While we were primarily interests in evaluating the reliability of the model in Equation 1 it is also possible to evaluate it in terms of the fit of the model to the data. We therefore evaluated the fit of the psychometric model in terms of *R*^2^ for tests of length 100, 300, and 500 trials. The fit was highly comparable for the theoretical (0.78, 0.91, 0.95) and empirical (0.75, 0.88, 0.94) data sets for the three test lengths.

^8^In reality, for a high reliability coefficient, it is also necessary that the test discriminates between participants. Thus, if we use a stimulus for which all participants always discriminate perfectly, there is no sampling error, but of course lack of a reliability coefficient because of the failure of the test to discriminate between participants.

^9^Virtually the same pattern holds when we perform these analyses on the empirical data rather than data simulated with the model.

^10^We also conducted all analyses using the NDE (or rather the ratio effect, NRE) as defined by Price et al. (2012) based on the slope across all stimulus levels. Results were similar to those presented below for both symbolic and for non-symbolic numeric distance.

^11^The nature of the adaptive task did not allow for the calculation of *P* or NDE measures.

^12^For some of these measures (*w*, numeric distance effect on RT), a small score suggests better ANS discrimination. For other measures (*P*, numeric distance effect based on *P*) a high (or less negative) score indicates better precision. To simplify interpretation of data, the sign of all correlations (and beta-weights) reported below were arranged so that a positive correlation means that superior performance on one measure is associated with superior performance on the other measure.

^13^We also performed a principal components factor analysis on all measures of ANS acuity (coded so that a positive value indicates better performance), the arithmetic fluency task and the inspection time task. This analysis revealed a three factor solution. The three simultaneous measures and arithmetic fluency had high positive loadings on the first factor, whereas NDE-RT loaded strongly but negatively on the same factor. We interpret this factor as arithmetic predisposition. The sequential test, symbolic NDE and the inspection time task all loaded positively on the second factor, which we interpret as a working memory/attention over time factor. Finally, the inspection time task also loaded in a third factor together with the NDE-P measure. This factor is harder to interpret. All factors had eigenvalues larger than 1, with a total of 64% explained variance, and all factor loadings were larger than 0.5.

^14^The term *predictive validity*, (or criterion-related validity) generally refers to the extent to which a measure allows discrimination between participants on basis of a behavioral criterion, as used here arithmetic fluency. It should be noted that a failure to find correlations with this criterion could mean either that the measure lacks in psychometric quality or that we are mistaken in our assumptions regarding the behavior that the measure should predict. Because several studies have established such correlations, and these associations are the main reason the research area has attracted so much recent interest, we have chosen to refer to this as “predictive validity,” in spite of the reasonable objections that could be raised against the use of the term.
